# Phase Structure and Properties of Ternary Polylactide/Poly(methyl methacrylate)/Polysilsesquioxane Blends

**DOI:** 10.3390/polym13071033

**Published:** 2021-03-26

**Authors:** Anna Kowalewska, Agata S. Herc, Joanna Bojda, Maria Nowacka, Mariia Svyntkivska, Ewa Piorkowska, Witold Kaczorowski, Witold Szymański

**Affiliations:** 1 Centre of Molecular and Macromolecular Studies, Polish Academy of Sciences, Sienkiewicza 112, 90-363 Lodz, Poland; asherc@cbmm.lodz.pl (A.S.H.); jbojda@cbmm.lodz.pl (J.B.); mnowacka@cbmm.lodz.pl (M.N.); mariiasv@cbmm.lodz.pl (M.S.); epiorkow@cbmm.lodz.pl (E.P.); 2 Institute of Materials Science and Engineering, Lodz University of Technology, Stefanowskiego 1/15, 90-924 Lodz, Poland; witold.kaczorowski@p.lodz.pl (W.K.); witold.szymanski@p.lodz.pl (W.S.)

**Keywords:** PLA/PMMA blends, ternary polymer blends, polysilsesquioxanes

## Abstract

Ternary blends of polylactide (PLA, 90 wt.%) and poly(methyl methacrylate) (PMMA, 10 wt.%) with functionalized polysilsesquioxanes (LPSQ-R) were obtained by solution blending. R groups in LPSQ containing hydroxyethyl (LPSQ-OH), methylglycolic (LPSQ-COOMe) and pentafluorophenyl (LPSQ-F5) moieties of different chemical properties were designed to modify PLA blends with PMMA. The effect of the type of LPSQ-R and their content, 1–3 wt.%, on the structure of the blends was studied with scanning electron microscopy (SEM) combined with energy dispersive spectroscopy (SEM-EDS), dynamic mechanical thermal analysis (DMTA) and Raman spectroscopy. Differential scanning calorimetry (DSC) and tensile tests also showed various effects of LPSQ-R on the thermal and mechanical properties of the blends. Depth-sensing indentation was used to resolve spatially the micro- and nano-scale mechanical properties (hardness and elastic behaviour) of the blends. The results showed clearly that LPSQ-R modulate the structure and properties of the blends.

## 1. Introduction

Biodegradable poly(lactic acid) (PLA) exhibits high strength and stiffness. Brittleness, low toughness, low melt strength, slow crystallization rate and low heat deflection temperature largely limit its wide use [1,2,3]. PLA may be toughened using various methods (plasticization, copolymerization, filling and melt blending with other polymers) and the relationships between PLA-based polymer blends composition, their morphology, mechanical properties and toughening mechanisms behind the observed phenomena have been thoroughly studied [4,5,6,7]. The interfacial compatibility on a nanoscale level and the phase behaviour have a decisive influence on polymer blends. Miscibility may be favoured when some special interactions, stronger than van der Waals forces (ionic interactions, dipole-dipole, donor-acceptor and weak hydrogen bonding) exist between the blended materials. The intrinsic properties of the components, as well as the characteristic morphology of the blend determine the improvement of the final properties, e.g., mechanical, thermal and barrier properties.

Interesting results were obtained on formation of miscible/immiscible binary blends containing poly(methyl methacrylate) (PMMA). PMMA is known for intrinsic high mechanical properties, UV resistance and long-term durability. Full miscibility of PLA and PMMA was achieved through solution precipitation [8,9] and melt blending [10] whereas solution casting resulted in two separate phases (co-continuous or separated inclusions) [8,10,11]. The immiscibility–miscibility transition behaviour of PLA/PMMA blends changes with composition [12,13] and temperature [14]. Rheological, thermomechanical, and mechanical properties of PLA/PMMA miscible blends with different compositions were extensively examined. The role of entanglement network in rheological and thermomechanical properties was explained [15,16,17,18,19,20]. The small size of the dispersed inclusions as well as the strong interfacial interactions in PLA/PMMA blends is responsible for increased tensile strength. PMMA was also shown to provide increased hydrolytic and structural stability to the PLA/PMMA blends [21,22,23]. However, the improvement of impact toughness in binary blends requires addition of quite large amount (>20 wt.%) of PMMA, which has an adverse effect on the compostability of PLA. In addition, the improvement of impact toughness may be often accompanied by a significant reduction of the tensile strength.

Ternary blends are much more complex systems with a wide range of possible microstructures. Many variables that influence the morphology, and properties include the volume fraction, inherent properties of the components and their miscibility. In the immiscible blends the interfacial tension between components can be efficiently reduced through compatibilization, which results in a better dispersion of a minor component and improved interfacial adhesion. That may prevent premature fracture resulting from formation of large cavities inside a minor component inclusions or due to separation of the components. Ternary PLA/PMMA blends with other thermoplastic polymers can be promising materials. The compatibilizers should be miscible with at least one component of the blend. Addition of copolymers containing functionalized methacrylate blocks is an effective way to achieve good compatibility and toughening [24,25,26,27,28,29,30,31,32,33,34]. Nanofillers, e.g., nanosilica [17,35] or other minerals [36,37], also have a positive effect on properties of PLA/PMMA blends. It is worth mentioning that PMMA was also applied as an effective compatibilizer that provided a toughening effect in blends of PLA/polyvinylidene fluoride [38], PLA/polybutadiene-g-poly(styrene-co-acrylonitrile) [39,40], PLA/poly(butylene adipate-co-butylene terephthalate) [41], PLA/polystyrene [42] or PLA/poly(epichlorohydrin-co-ethylene oxide) [43].

**Scheme 1:**
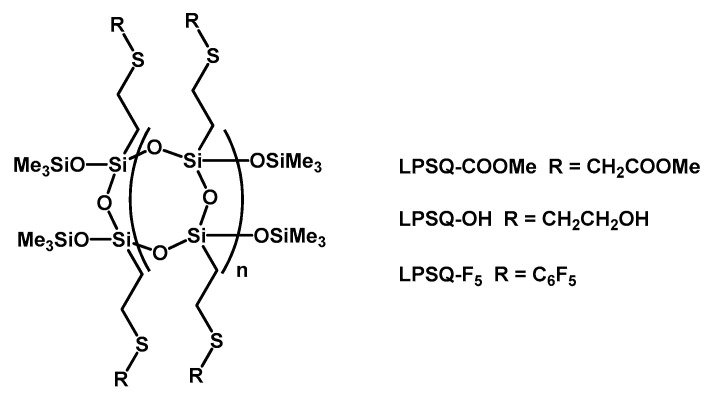
Chemical structure of (poly[2-(hydroxyethylthio)-ethylsilsesquioxane] (LPSQ-OH), poly[2-(methylthioglycolate)-ethylsilsesquioxane] (LPSQ-COOMe) and poly[2-(pentafluorophenylthio)-ethylsilsesquioxane] (LPSQ-F5).

We have recently found that linear, ladder-like polysilsesquioxanes (LPSQ-R) (Scheme 1) may be applied as interesting modifiers of PLA [44,45,46,47]. LPSQ-R are amorphous substances of low glass transition temperatures that are partially miscible with PLA [44,45,46]. Their net effect may be attributed to plasticization and dispersion in PLA matrix and supramolecular interactions that involve side functional groups R [6,44,45,46]. In this report, we focus on the influence of selected LPSQ-R on PLA/PMMA (90:10) blend. The composition allowed for studying dispersion of PMMA particles within PLA matrix. Moreover, this blend exhibited good ductility.

Phase structure and dispersion of PMMA in PLA matrix was studied with scanning electron microscopy (SEM) and Raman mapping. The observed differences were correlated with the bulk thermal and tensile properties of the blends as well as surface nanomechanical characteristics and wettability. The obtained results show that small amounts of functionalized polysilsesquioxanes may improve the dispersion of PMMA in PLA and modulate the properties of such ternary blends. Although polysilsesquioxanes are not biodegradable, they are non-toxic and biocompatible as other silsesquioxanes [48].

## 2. Materials and Methods

### 2.1. Materials

Ladder-like polysilsesquioxanes LPSQ-R (Scheme 1) were prepared as previously described [44,45]. The precursor used for their synthesis was poly(vinylsilsesquioxane) of *M*_n_ = 1 kg mol^−1^, *M*_w_/*M*_n_ = 1.4. PLA used in the studies was a commercially available NW4032D grade from NatureWorks LLC, Minnetonka, MN, USA (*M*_w_ = 130 kg mol^−1^, *M*_w_/*M*_n_ = 1.9) containing 1.2 mol% of D-lactide units. Poly(methyl methacrylate) was Altuglas BS 572 Clear (Resinex, Warsaw, Poland, *M*_n_ = 179 kg mol^−1^, *M*_w_/*M*_n_ = 2.4, *T*_g_ = 119.5 °C). Solvents used for the synthesis and preparation of blends (THF, CH_2_Cl_2_, Krakchemia S.A., Kraków, Poland) were purified following literature procedures [49].

Blends of PLA and PMMA containing LPSQ-R modifiers (10, 20 or 30 wt.% with respect to the amount of PMMA) were prepared by solution blending (Table 1). The blends are referred through this paper as, for example B-R-10, where R indicates the type of functional group, and the number stands for the LPSQ-R weight content with respect to PMMA. For comparison, PLA blend with 10 wt.% of PMMA was also prepared.

In general, the PLA/PMMA mixture (9.0 g and 1.0 g, respectively) was dissolved in CH_2_Cl_2_ (68 mL), to obtain 10 wt.% solution. After 24 h, a specific volume of LPSQ-R solutions in CH_2_Cl_2_ (3 wt.% LPSQ-COOMe or LPSQ-F5; corresponding to 0.1 g in 2.4 mL of the solvent for B-R-10) or THF (4 wt.% LPSQ-OH; corresponding to 0.1 g in 2.7 mL of the solvent for B-R-10) was added dropwise. The mixtures were stirred magnetically for 2 h at room temperature (RT). The mixtures containing LPSQ-OH became cloudier in a short time after the addition of the polysilsesquioxanes and remained turbid on prolonged stirring. The effect was not observed for LPSQ-COOMe and LPSQ-F5. No increase of viscosity of the mixtures was observed. The prepared compositions were then poured into Petri dishes and left for free solvent evaporation. The obtained samples were not tacky. Solid products were dried under high vacuum (0.01 Torr) for 24 h at 80 °C, then for 24 h at 100 °C, and studied for their thermal stability with thermogravimetric analysis. For further studies, 0.5 and 1 mm thick films were compression moulded at 190 °C and rapidly quenched between metal blocks held at 0 °C to hinder crystallization of PLA matrix. Differential scanning calorimetry (DSC) measurements evidenced that all the prepared films were amorphous.

### 2.2. Analytic Methods

Morphological investigations were carried out for amorphous films of neat PLA, PMMA, PLA/PMMA (B-0) blend and ternary PLA/PMMA/LPSQ-R blends that were cryo-fractured, sputtered with gold using a Jeol Fine Coater 1200 (JEOL, Tokyo, Japan) and analysed with scanning electron microscopy (SEM) using SEM Jeol 6010LA (JEOL, Tokyo, Japan) operating in the high vacuum mode at an accelerating voltage of 10 kV. The distribution of silicon atoms of LPSQ-R in the studied samples was examined with SEM with energy dispersive spectroscopy (SEM-EDS). Prior to the examination, the surfaces were sputtered with carbon using a coater Q150R ES (Quorum Technologies, Lewes, UK). To have a better insight into the structure, films of selected materials were enzymatically etched according to [50,51]. The solution of Proteinase K (4 mg), Trizma base (61 mg) and sodium azide (2 mg), all from Sigma Aldrich (Munich, Germany), in distilled water (5 mL), was prepared. The samples were etched in the solution at 37 °C for 1–2 h, washed with a distilled water, dried, sputtered with gold and examined by SEM.

Dynamic mechanical thermal analysis (DMTA) was carried out on 1 mm thick rectangular specimens, 17.5 mm × 12 mm, in a single cantilever bending mode, using a DMTA TA Q-800 Thermal Analyser (TA Instruments, New Castle, DE, USA) at a frequency of 1 Hz and a heating rate of 2 °C min^−1^ from −100 to 140 °C.

Raman signal (spectra and maps) were collected with an InVia Raman microscope (Renishaw plc, Gloucestershire UK). The polymer surfaces were analysed using a 532-nm laser with 20× objective lens (Carl-Zeiss Jena, Germany), providing approximately 25 mW of laser power at the sample. Single spectra were collected with an exposure time of 20 s in the spectral collection range 700–1800 cm^−1^. 2-D Raman maps were collected from 200 μm × 100 μm ^2^ areas with 10 µm spatial resolution. All operations on the data (baseline correction, normalization) and direct classical least squares (DCLS) component analysis were performed using Wire 5.1 application. For better visualization, the maps appearance was interpolated.

Phase transitions in the studied samples were detected using a DSC 2920 (TA Instruments, New Castle, DE, USA). Thermograms were recorded for samples of blend cast films heated in N_2_ atmosphere at a rate of 10 °C min^−1^ from 20 to 190 °C. After the first heating, the samples were kept at 190 °C for 5 min and then cooled down to ensure the same thermal history. The phase transitions were studied during the second heating, at 5 °C min^−1^. The degree of crystallinity (*X*_c_) was estimated from the enthalpies of cold crystallization exotherms and melting endotherms using Equation (1):

(1) $$X_{c}=\frac{\Delta H_{m}\cdot 100\%}{\Delta H_{0}}$$

where Δ*H*_m_ and Δ*H*_0_ are the enthalpy of fusion of PLA in the blends and the crystalline phase of PLA (93 J/g, enthalpy of melting of *α*-crystals [52]), respectively.

Thermogravimetric analyses of the studied polymeric materials were carried out with a Hi-Res TGA 2950 Thermogravimetric Analyzer (TA Instruments, New Castle, DE, USA) in N_2_ atmosphere (heating rate of 10 °C min^−1^, resolution 3, sensitivity 3).

Tensile properties were measured using Linkam Tensile Stress Testing System TST 350 (Linkam Scientific Instruments Ltd., Waterfield, UK). At least five 0.5 mm thick oar-shaped specimens, with 3.81 mm gauge length and gauge width of 1.59 mm, were drawn to fracture at a rate of 5% min^−1^, at 25 °C. The average values of mechanical parameters were calculated. Selected specimens were analysed after fracture with polarized light microscopy (PLM) using microscope Nikon Eclipse 80i (Nikon, Tokyo, Japan).

Nanomechanical properties on the surface and at the cross-section of the compression moulded 0.5 mm thick polymer films were determined with a G-200 Nano Indenter (MTS Nano Instruments/KLA-Tencor Corporation; Milpitas, CA, USA) equipped with TestWorksPro 4 software. The impressions were made with a Berkovich type indenter with a half angle equal to 65.3° and a radius of roundness. The data analysis with the Analyst program was based on Oliver and Pharr’s approach [53]. The studies were carried out using two methods: CSM (Continuous Stiffness Measurement) and BASIC [54]. The CSM method is based on continuous measurement of the stiffness (*S*) during the material penetration to estimate hardness (*H*) and modulus of elasticity (*E*) of the material as a function of penetration depth (*h*). The test was used to measure the mechanical properties of the specimens from the front with 9 prints per sample (penetration depth 1500 nm). BASIC method was used to map the mechanical properties from the front at the maximum load point. Maps of *E* and *H* were obtained with 100 indents in a 10 × 10 matrix with a step of 20 μm.

Surface free energy was estimated for the compression moulded 0.5 mm thick films by contact-angle measurements (sessile drop technique) at the film–air interface, as described earlier [46], using deionized water and glycerol (Chempur, Piekary Slaskie, Poland; pure p.a., anhydrous) as the reference liquids. Static and advancing contact angles of sessile droplets of reference liquids at the film–air interface were measured right after the deposition at RT with a Ramé-Hart NRL contact-angle goniometer (Ramé-Hart Instrument Co., Succasunna, NJ, USA) equipped with a video camera JVC KYF 70B (Yokohama, Japan) and drop-shape analysis software (“Drop”, version 2.1, written and authored by Dr. hab. Stanisław Sosnowski at Centre of Molecular and Macromolecular Studies, Polish Academy of Sciences). The surface free energy (including the polar and dispersive components) was estimated by the Owens–Wendt method [55]. The values of contact angles are averages of at least four measurements taken on different areas of the same sample and calculated with their standard deviations. The contact angle for each drop was calculated as the arithmetic mean of the left and right angles.

## 3. Results and Discussion

### 3.1. Phase Distribution in the PLA/PMMA Mixture and Ternary Blends Containing LPSQ-R

The application of linear double strand polysilsesquioxanes as modifiers in PLA/PMMA blends is related to our previous works on PLA/LPSQ-R systems [44,45,46]. Linear polysilsesquioxanes have a specific double strand backbone, which makes them much less flexible than linear polysiloxanes and prevents chain coiling. The presence of functional groups at each of the silicon atoms increases effectiveness of interactions, e.g., in supramolecular systems. The functionalized LPSQ-R of similar molecular weight selected for this study have low glass transition temperature (*T*_g_) (LPSQ-COOMe: −41 °C; LPSQ-OH: −46 °C; LPSQ-F5: −10 °C) [45,46]. We have previously showed that LPSQ-COOMe [45] and LPSQ-F5 [46] are well dispersed and partially miscible with PLA at c ≤ 5 wt.%. LPSQ-OH formed separated inclusions with sizes up to a few micrometers in the polyester matrix for lower contents of the additive, yet at 5 wt.% the maximum inclusion size increased to about 10 µm [45]. The structural differences may be correlated with the strength of interactions between the functional groups and PLA matrix. LPSQ-COOMe may form only rather weak hydrogen bonds with carbonyl groups (-CH_3_···O=C-). This phenomenon may be of importance as similar mechanisms are known to operate during the formation of PLA stereocomplex structures [56]. The contacts between LPSQ-F5 and PLA were explained by the formation of complexes similar to n→π* attractive interactions. Both LPSQ-COOMe and LPSQ-F5 could act as effective modifiers of PLA drawability and tensile toughness without a significant reduction of *T*_g_ of PLA [45,46]. Moreover, enhancement of low temperature nucleation, which intensified cold crystallization, was observed. In the present study, PLA/PMMA blends modified with LPSQ-R were prepared by solution blending. It is known that miscible PLA/PMMA blends are most readily formed by melt blending while solution blending results in phase separation [12]. Nevertheless, this obstacle helped to demonstrate clearly the effect of LSPQ-R. The low weight ratio of PMMA to PLA was also chosen to better illustrate the changes in the components’ distribution. It should be stressed that the effective content of LPSQ-R in the blends is very low (≤3 wt.%).

**Figure 1:**
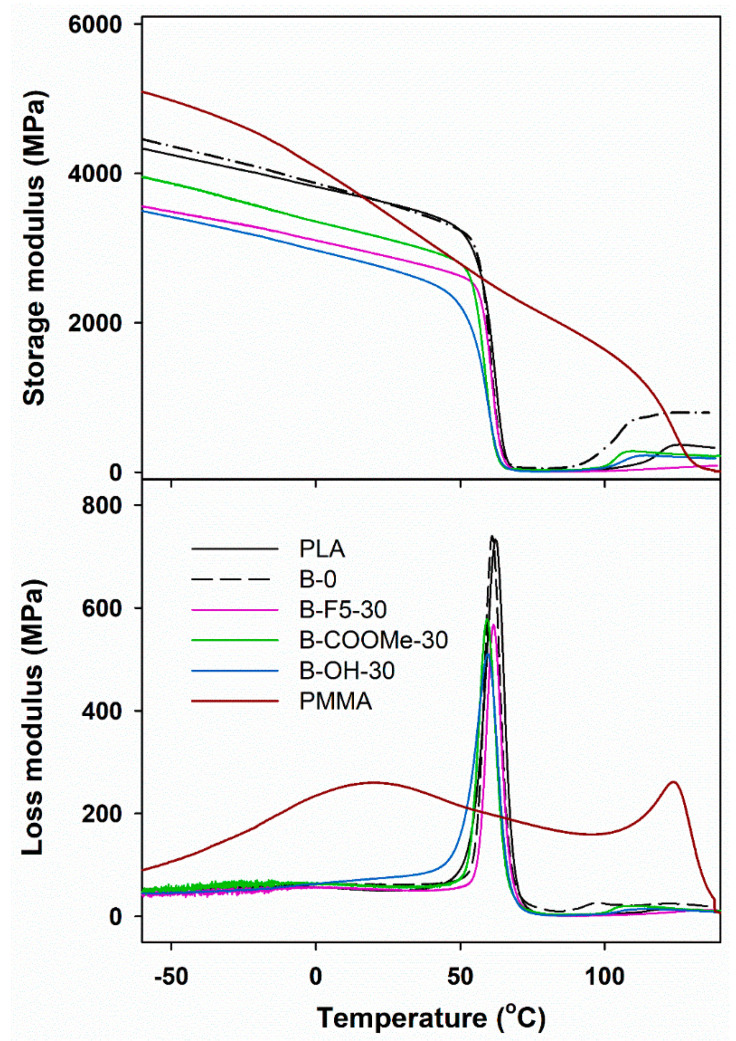
Storage modulus (*E’*) and loss modulus (*E″*) of neat polylactide (PLA), poly(methyl methacrylate (PMMA), PLA/PMMA blend (B-0) and selected LPSQ-R modified blends vs. temperature.

Figure 1 shows the DMTA results, the storage modulus (*E’*) and loss modulus (*E″*) of PLA, PMMA, PLA/PMMA blend (B-0) and the blends with the 3 wt.% of LPSQ-R. The *E″* peaks of PLA and PMMA, with maxima at 62 °C and at 124 °C, correspond to the glass transition in these polymers. In addition, *E″* plot of PMMA exhibits a broad β-peak with a maximum at 20 °C, correlated with the mobility of ester side groups with respect to the main chain and overlapping the α-peak corresponding to the glass transition. *E’* of PMMA and *E’* of PLA decreased with increasing temperature and dropped above 100 °C and above 50 °C, respectively, in the glass transition regions of these polymers. The pronounced *E″* peak of PLA/PMMA blend at 61 °C corresponded to the glass transition in PLA. In the glassy region, *E*″ of the blend was slightly higher that of PLA because of PMMA β-relaxation contribution. The result suggested that the PLA/PMMA blend was immiscible. The glass transition of PMMA in the blend did not show up because of the small PMMA content and a strong decrease of stiffness of the blend above PLA glass transition that made correct measurement impossible. *E″* plots of B-OH-30, B-F5-30 and B-COOMe-30 were similar to that of PLA/PMMA blend, with *E*’ peak temperature of 60, 61 and 59 °C, respectively. The small decrease of *E″* peak temperature resulted from dispersion of LPSQ-R fraction in PLA on a molecular level, as it was observed previously for PLA blends with LPSQ-R [45,46]. This conclusion was corroborated by the results of SEM EDS analysis.

*E’* plot of PLA/PMMA blend resembled that of PLA. *E’* of the blend was slightly higher than *E*′ of PLA in the low temperature range and slightly lower above 0 °C, and it finally dropped in the glass transition region. *E*’ temperature dependencies of B-OH-30, B-F5-30 and B-COOMe-30 resembled that of PLA/PMMA blend, except for lower *E’* values in the glassy region caused by the presence of LPSQ-R.

**Figure 2:**
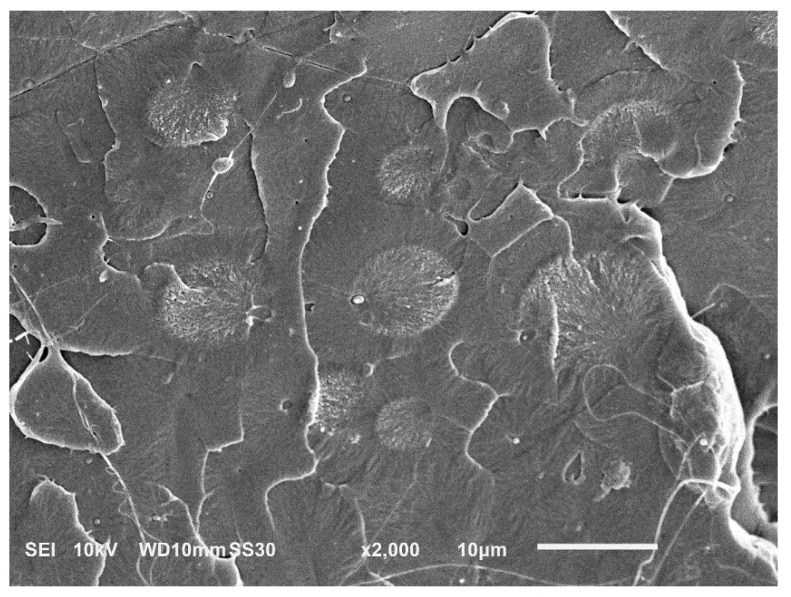
Scanning electron microscopy (SEM) micrograph of cryo-fracture surface of PLA/PMMA blend.

**Figure 3:**
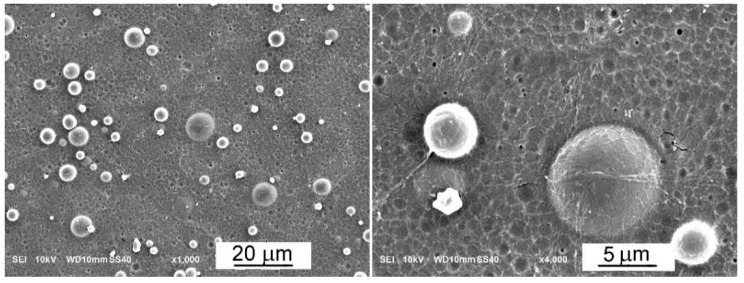
SEM micrographs of PLA/PMMA blend film etched for 1 h.

The blend structure was studied by SEM. The results of SEM analysis, summarized briefly in Table S1, evidenced phase separation in the blends. SEM micrographs (Figure 2) of the cryo-fracture surface of PLA/PMMA (B-0) blend reveal the presence of oval areas, with sizes of several micrometers, where fracture propagated differently than in the surrounding matrix, which is suggestive of phase separated inclusions. Figure 3 shows the etched surface of this blend film. Proteinase K is known to degrade L-lactide units, hence the etching should expose PMMA inclusions. Indeed, on the etched surface particles of various sizes are seen, some laying on the surface and some still embedded in the matrix. The sizes of the large particles correspond to the sizes of the oval areas visible on the fracture surface in Figure 2 that confirms that the latter are PMMA inclusions. It is worth noting that after longer etching, the sizes of the visible particles somewhat diminished, except for those still embedded in matrix. This suggest that the PMMA particles could contain a fraction of PLA, despite the lack of DMTA evidence.

**Figure 4:**
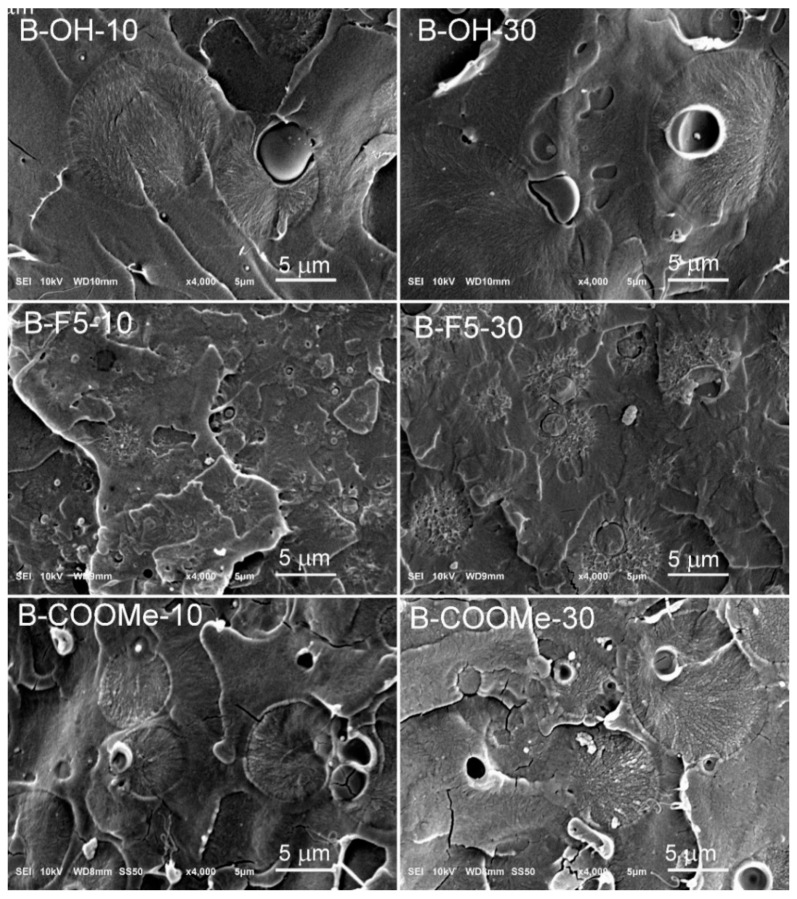
SEM micrographs of cryo-fracture surfaces of PLA/PMMA blends modified with LPSQ-R.

**Figure 5:**
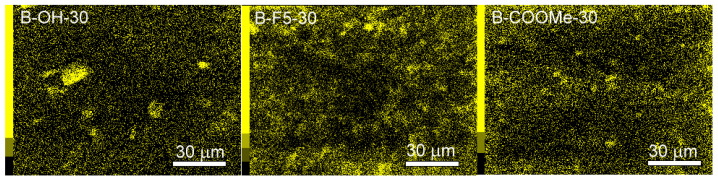
SEM EDS Si mapping of PLA/PMMA blends modified with LPSQ-R.

SEM micrographs of surfaces of cryo-fractured ternary blends with 1 and 3 wt.% of LPSQ-R are shown in Figure 4. On the surfaces oval areas are visible, where the fracture propagated differently than in the surrounding polymer, as in PLA/PMMA blend in Figure 2, and identified as PMMA inclusions. However, in the micrographs, also other phase separated particles are present, with sizes dependent on the additive type and content. They were accompanied with holes of similar sizes that were inhabited by the inclusions before the fracture. However, Si mapping of the blends with 3 wt.% of LPSQ-R, presented in Figure 5, confirms that LPSQ-R formed inclusions in the blends but were also dispersed in the surrounding polymers. Previously, we have found that PLA blends with LPSQ-R were phase separated but partially miscible [44,45,46]. In the present study, the phase separated LPSQ-R rich inclusions did not show up in DMTA results, most possibly because of their small content. SEM analysis of cryo-fracture surfaces of the blends with LPSQ-OH also demonstrated the presence of large LPSQ-OH-rich inclusions of sizes of several micrometers, irrespectively of the additive content. Although they were dispersed in the PLA rich matrix, many of them were embedded, in whole or in part, in PMMA particles. In the ternary blends with LPSQ-F5 and LPSQ-COOMe, the largest additive rich inclusions were markedly smaller than in the materials with LPSQ-OH. Although the largest inclusion size increased with increasing additive content, it did not exceed 3 µm in B-F5-30. In the blends with LPSQ-COOMe, the sizes of the largest inclusions were similar. As shown in Figure 4, in both types of ternary blends, numerous additive rich inclusions were located in the PMMA particles. SEM EDS Si mapping did not show any depletion of Si around LPSQ-R rich inclusions; hence, LPSQ-R were not only dispersed on a molecular level in PLA but also in PMMA.

**Figure 6:**
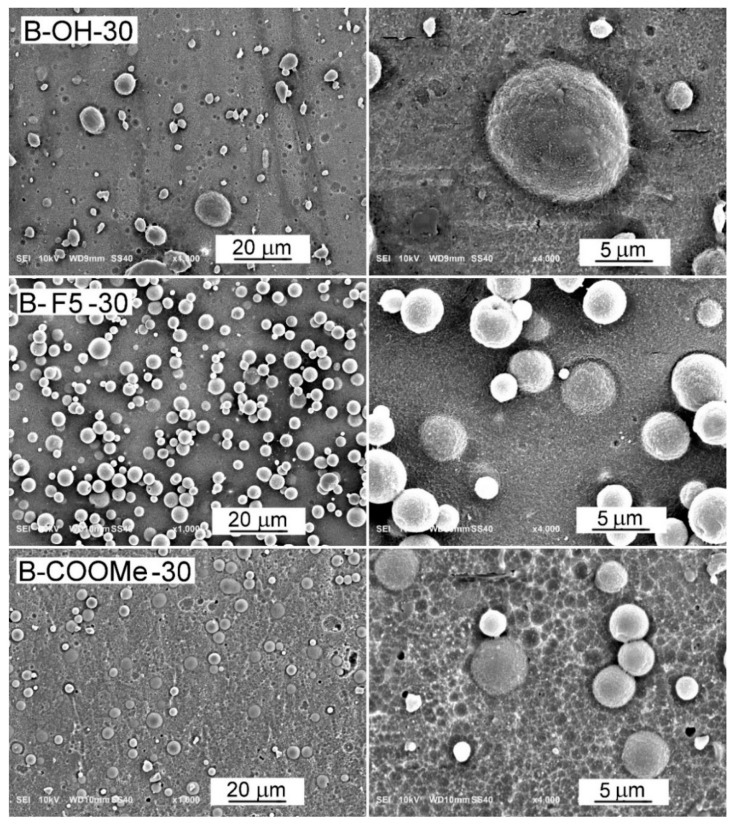
SEM micrographs of etched PLA/PMMA blends modified with 3 wt.% of LPSQ-R.

Figure 6 shows SEM micrographs of the blends with 3 wt.% of LPSQ-R after 2 h etching. Similarly to PLA/PMMA blend, numerous particles are visible, located on the surface or still embedded in the polymer. The small particles in B-OH-30 were accompanied by large ones, with diameters exceeding 10 µm. In contrast, sizes of particles in B-F5-30 and B-COOMe-30 were more uniform, up to several micrometers. Judging from their size, the particles might be PMMA or LPSQ-R rich particles. However, as seen in Figure 5, numerous LPSQ-R particles were located inside PMMA. Moreover, LPSQ-R are amorphous substances with low *T*_g_, unable to retain their shape at RT. Therefore, the particles shown in Figure 5 are rather PMMA-rich particles.

**Figure 7:**
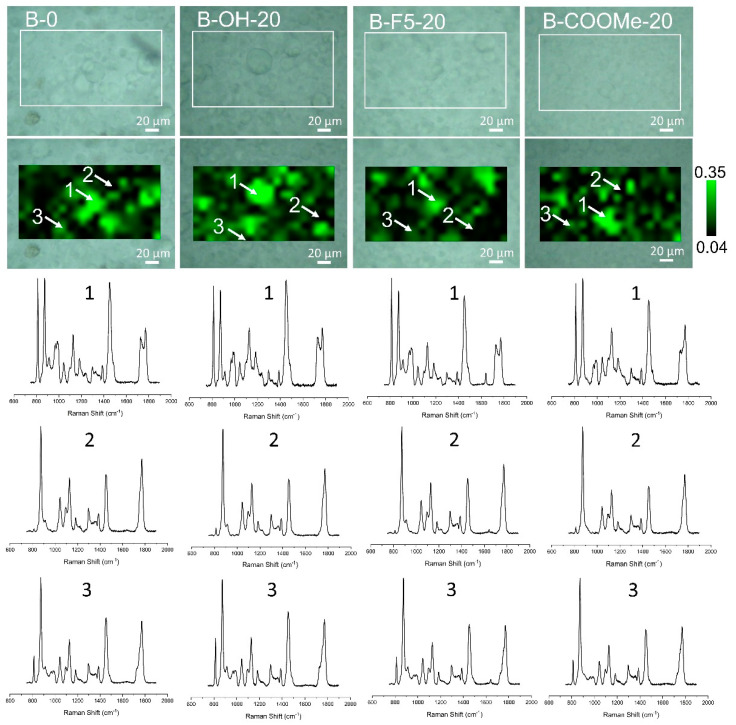
Raman maps of PLA/PMMA and ternary blends with 2 wt.% content of LPSQ-R illustrating distribution of PMMA in the blends (with corresponding optical micrographs in the background) and spectra of selected regions.

Raman spectroscopy mapping was used to detect changes of concentration of PMMA in the studied samples (Figure 7 and Figures S1–S4, Table S2). Very clear data were obtained, and evaluation of the main components’ dispersion was possible. Raman spectra of PLA and PMMA differ in the position of *ν*(C=O) mode (1767 cm^−1^ for PLA and 1726 cm^−1^ for PMMA) and the presence of *ν*(C-COO) (873 cm^−1^, PLA), *ν*(CH_2_) (853 cm^−1^, PLA) and O–CH_3_ rock (990 cm^−1^, PMMA) vibrations.

Comparative analysis of the relative intensity of those bands can be indicative of the blending effectiveness. Raman maps evidenced phase separation in the blends; PMMA formed inclusions, as already shown by SEM, although it may be also partly dispersed in PLA. The size of inclusions seems to decrease on increasing concentration of LPSQ-OH. Better dispersion was observed for LPSQ-COOMe. PMMA was well dispersed within PLA even at a very low content of this additive (B-COOMe-10). LPSQ-F5 is an intermediate—rather good dispersion was noted for B-F5-20. The maps illustrating distribution of PMMA corroborate the structure of blends shown in optical micrographs (Figure 7 and Figures S1–S4) and microstructures shown in Figure 6. Samples containing LPSQ-COOMe are the most uniform among those materials. Unfortunately, the intensity of characteristic Raman vibrations of LPSQ-R was below the detection limit.

The structural differences of the studied PLA/PMMA/LPSQ-R blends may be explained in terms of supramolecular interactions characteristic of R groups. Side groups of LPSQ-OH act as donors of hydrogen bonds to oxygen atoms of carbonyl groups in both PLA and PMMA. That is why it appears to be worse dispersed in the blend polymers. Methylglycolic moieties of LPSQ-COOMe are rather inert, although they can act as acceptors/donors of weak -CH_3_···O=C- hydrogen bonds. LPSQ-F5 is a specific case—as it was mentioned, pentafluorophenyl species may form weak complexes with carbonyl groups, which may bring about attractive interactions between LPSQ-F5 and both PLA and PMMA.

### 3.2. Thermal and Mechanical Properties of the Hybrid Blends

**Figure 8:**
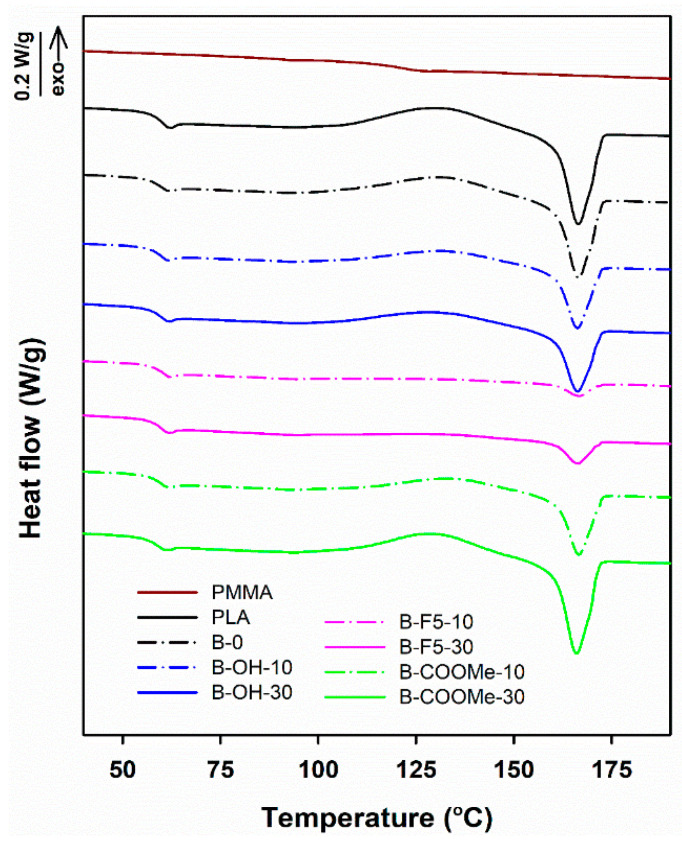
DSC thermograms of neat PLA, PMMA, PLA/PMMA and PLA/PMMA blends modified with LPSQ-R recorded during the second heating at 5 °C min^−1^.

The thermal properties of the blends were examined with DSC. DSC first heating thermograms of cast films collected in Figure S5 show melting endotherms with peak temperatures (*T*_m_) at 166–167 °C and melting enthalpies (Δ*H*_m_) ranging from 40 to 50 J/g. The lack of cold crystallization exotherms evidenced that crystallization in the films was accomplished during preparation. During subsequent cooling from 190 °C no crystallization was observed, as shown in Figure S6; thus, the cooled films were amorphous and able to crystallize during second heating. Preliminary experiments demonstrated that during the second heating at 10 °C min^−1^ only weak cold crystallization occurred, followed by melting, and thus the phase transitions in the blends was studied at a decreased heating rate of 5 °C min^−1^. The second heating thermograms of the neat polymers and the blends are collected in Figure 8. The PMMA thermograms shows only glass transition with *T*_g_ of 120 °C. In turn, for neat PLA, above the glass transition with *T*_g_ at 60 °C, the cold crystallization exotherm was visible, with the peak temperature *T*_cc_ at 131 °C and cold crystallization enthalpy (Δ*H*_cc_) of 29 J/g, corresponding to 31 wt.% crystallinity. The cold crystallization was followed by melting with *T*_m_ at 166 °C and Δ*H*_m_ equal to Δ*H*_cc_. The thermal behaviour of PLA/PMMA blend was similar with nearly the same *T*_cc_^,^ *T*_m_^,^ and Δ*H*_m_ of PLA component. Due to overlapping of cold crystallization exotherm of PLA and glass transition of PMMA, only Δ*H*_m_ allows to estimate correctly the crystallinity of PLA in the blends. It is worth noting that crystallization of PLA was restricted in the presence of PMMA in their miscible blends [20,57]. PMMA retarded also the crystallization of PLLA plasticized by PEO by reducing the chain mobility in the blends [58,59]. In the present study, the phase separated structure of the PLA/PMMA blend resulted in the lack of significant influence of PMMA on PLA crystallization in the blend. Similarly to PLA/PMMA, thermograms of the ternary blends exhibited the glass transition, with *T*_g_ of 59–60 °C, and the cold crystallization exotherms followed by melting endotherms. The slight enhancement of cold crystallization due to the addition of LPSQ-R was observed only in the blends with 2 and 3 wt.% of LPSQ-COOMe, reflected in a decrease of *T*_cc_ to 125–126 °C and an increase of Δ*H*_m_ to 31–32.5 J/g _PLA_, which corresponds to 33.5–35 wt.% crystallinity. The significant enhancement of the cold crystallization of PLA in the blends with LPSQ-COOMe was observed by us previously [44,45]. However, in the LPSQR-COOMe modified PLA/PMMA blends, the dispersion of the additive was much worse, and its content in PLA was smaller due to location of inclusions in PMMA rich domains. Despite the differences in crystallinity developed during cold crystallization, the melting behaviour of all the blends was similar to that of PLA, and their *T*_m_ values were practically the same, at 166–167 °C. It is worth noting that the cooling rate of 10 °C min^−1^ was sufficient to cool the blends to the glassy state without crystallization. Thus, one can expect that the compression moulded films of the blends quenched between metal blocks were also amorphous.

The thermal stability of the blends was studied with TGA. The prepared PLA/LPSQ-R were thermally stable, and the additives did not change the structure of PLA backbone (Figure S7). The main decomposition of PLA was noted at the temperature of peak weight loss derivative *T*_d_ = 354.2 °C at a rate of weight loss of 9.5%∙min °C^−1^, and leaving char residue of 1.2 wt.% at 600 °C. The B-0 and blends with LPSQ-R were more thermally stable (*T*_d_ = 360–365 °C, irrespectively of the kind of R and the content of LPSQ-R). The rate of decomposition was the same as that of neat PLA, and the char residue was negligible. The only exception was the char left after decomposition of B-OH blends that increased to about 2 wt.%, which corroborates earlier reported observations [44]. The hydroxyethyl groups may take part in thermally induced process of redistribution of ester bonds, which would result in incorporation of LPSQ-OH in PLA matrix.

**Figure 9:**
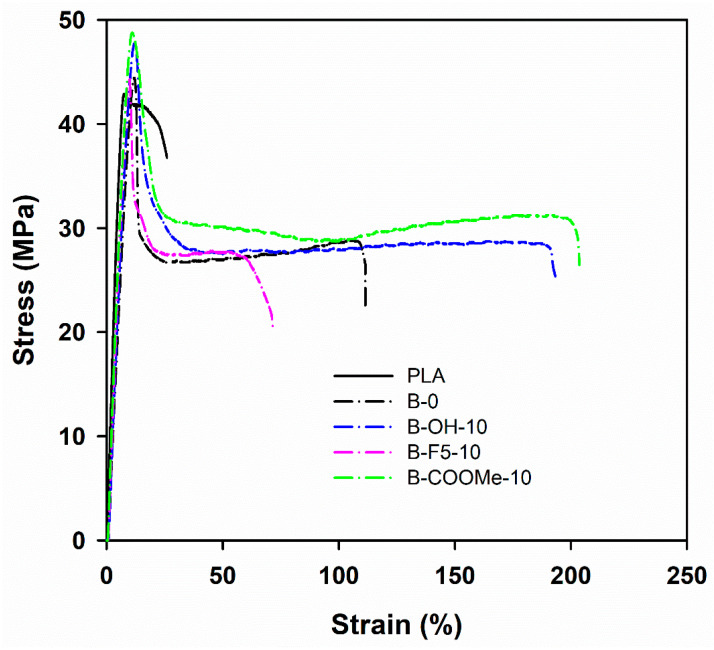
Engineering stress-engineering strain dependencies of PLA, PLA/PMMA and PLA/PMMA blend modified wit 1 wt.% of LPSQR, recorded during drawing at 5%/min.

Mechanical properties of the obtained blends were studied by tensile testing. Exemplary engineering stress-engineering strain dependencies of the materials examined are shown in Figure 9, whereas average values of mechanical parameters are collected in Table S3. PLA exhibited the yield stress (*σ*_y_) close to 44 MPa and fractured at the elongation (*ε*_b_) and stress (*σ*_b_) of 27% and 42 MPa, respectively. PLA/PMMA blend exhibited slightly higher *σ*_y_ of 46 MPa. Beyond the yield, the stress decreased to about 28 MPa, and *ε*_b_ of 95% was reached, at the average *σ*_b_ of 28.5 MPa, indicating an improved drawability as compared to neat PLA. The stress–strain dependencies of all ternary blends also showed a decrease of stress beyond the yield, similarly to PLA/PMMA blend. Because of that, the yield strength of all blends was determined by *σ*_y_. However, only B-OH-10 and B-COOMe-10 exhibited significantly improved drawability in comparison to PLA/PMMA. Their *σ*_y_ values, 45 MPa and 48 MPa, respectively, were close to that of PLA/PMMA blend, but the average *ε*_b_ of about 160 and 190, respectively, was achieved at *σ*_b_ close to 28 MPa. With increasing LPSQ-OH content *ε*_b_ of the blends worsened dramatically, to below 15%, despite a decrease of *σ*_y_ to 39–40 MPa. The increase of LPSQ-COOMe content to 2 wt.% resulted in a decrease of *ε*_b_ to about 110% and to 50% at the 3 wt.% content. *ε*_b_ of the blends with LPSQ-F5 also worsened with increasing additive content, but even at 1–2 wt.% content, it was only about 70% and dropped to 11% at 3 wt.% content.

During drawing of all the blends, the necking was observed indicating the predominant deformation mechanism associated with shear bands. The neck region of PLA/PMMA blend did not exhibit a significant loss of transparency. PLM micrograph of this region (Figure S8) showed crazes but rather short and distorted by later formation of shear bands. We hypothesize that the propagation of crazes, initiated on specimen surfaces, was terminated on PMMA inclusions, which prevented for early fracture and enabled the formation of shear bands. In the neck regions of the ternary blends exhibiting enhanced drawability, also short and distorted crazes were seen, as shown in Figure S9. However, the intense whitening was observed in the neck regions of these blends, indicating the occurrence of cavitation. Cavitation in PLA based blends, inside the inclusions of minor component or at the interface, was observed also by others [4,6,60]. The shear yielding of PLA matrix triggered by the cavitation was identified as the main toughening mechanism [4,6]. Thus, cavitation related to LPSQ-R inclusions could improve the drawability of the LPSQ-R modified blends in comparison to PLA/PMMA, although the increase of LPSQ-R content causing an increase of inclusion size and number could result in a premature fracture.

### 3.3. Mechanical Properties in Micro-Scale and Surface Characteristics

**Figure 10:**
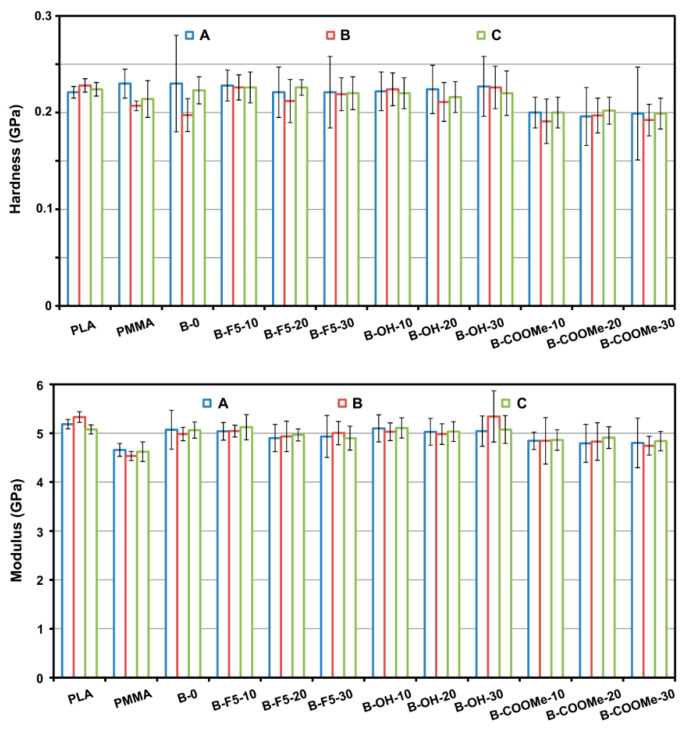
Average mechanical properties (hardness *H* and elastic modulus *E*) in the studied samples (A: along the surface B: cross-section at the edge; C: cross-section, middle area).

**Figure 11:**
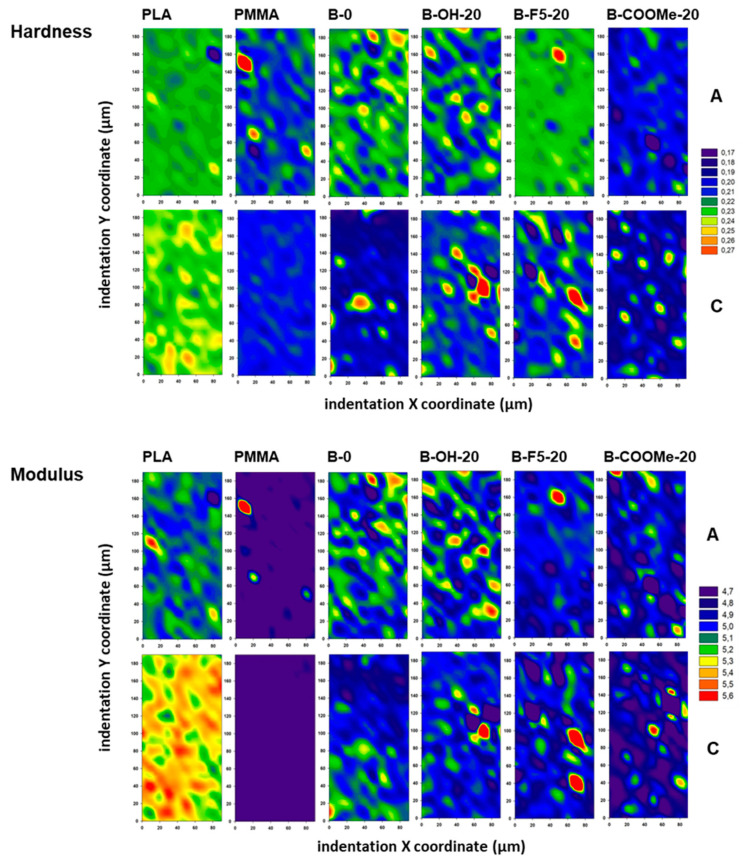
Maps of nanomechanical properties (hardness *H* and elastic modulus *E*) in compression moulded samples of the neat components and selected blends (A: surface area; C: cross-section, middle area).

The extracted data allowed to evaluate mechanical properties of the blends along their surfaces and cross sections (Figure 10 and Figure 11; Figures S10 and S11). Nanoindentation experiments showed that the hardness of PLA, PMMA, B-0 and the blends with LPSQ-OH and LPSQ-F5 is similar while the average value of *H* for B-COOMe decreased by about 10% irrespectively of the amount of polysilsesquioxane. The elastic modulus *E* remained unchanged at the level characteristic of PLA in the less homogenous blends B-0, B-OH and B-F5. *E* of PMMA is slightly smaller, and it was reflected in the B-COOMe of rather uniform concentration of the components.

Hardness and modulus mapping using the BASIC method allowed for their quantitative determinations of the studied samples. All maps are prepared based on 200 indents with spacing 10 µm (single map includes 200 indents covering the 190 × 90 µm area). The spacing of the indents is selected taking into account the rules of minimum indentation spacing, which ensure that no deformations/stresses of one indent affect the neighbour indent. The minimum safety distance is assumed to be 20 times the indentation depth [61]. The depth of the indents for the tested samples was about 500 nm, i.e., the minimum distance between the indents should be 10 µm. Maps of nanomechanical properties (Figure 11 and ESI-10-11) were influenced by the mixture composition and correspond to the content of PMMA showed in Raman spectra (Figure 7 and Figures S2–S4). B-COOMe exhibit uniform distribution of *H* and *E.*

The decrease of elastic modulus and hardness seems to be related both to the intrinsic properties of PMMA and the character of the additive (viscous liquids) at RT. Samples containing LPSQ-OH and LPSQ-F5 were not softened, but contrary to the features showed in B-0 maps, the structure of samples is similar on the surface and at the cross-section. B-OH contain areas of increased *E* and *H*, which may be the result of local physical crosslinking by hydrogen bonds donated by hydroxyethyl groups of LPSQ-OH. We have recently showed that nanomechanical properties of PLA blends containing low molecular weight cyclotetrasiloxane analogues of LPSQ-OH were distributed rather uniformly while more pronounced local variations of *E* and *H* were observed (in accordance with SEM EDS) for samples containing cyclosiloxanes with carboxylic groups [62]. The changes of surface hardness and elasticity observed for B-OH indicate that the accumulated number of functional groups grafted to the polysiloxane backbone hinder blending of the components, which results in local change of properties.

**Figure 12:**
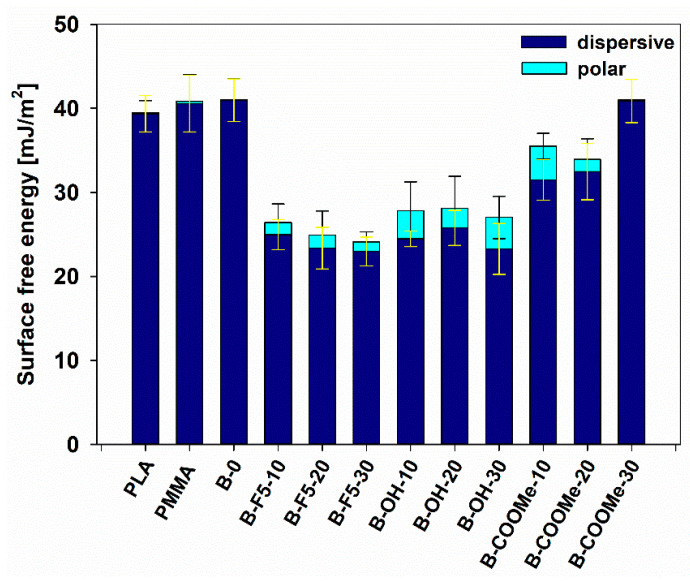
Changes of surface energy measured for native PLA, PMMA, their blend B-0 and blends with LPSQ-R.

We have also found that addition of functionalized LPSQ-R (R = OH, F5) to PLA/PMMA blend resulted in a slight decrease of surface free energy (*γ*_s_) with respect to the neat PLA and PLA/PMMA (B-0) (Figure 12). The decrease of *γ*_s_ does not seem to depend much on the amount of the additive. For B-F5, the most pronounced effect may be typically related to the presence of fluorine atoms. The explanation of the increase of hydrophobicity in B-OH is not intuitive. The inhomogeneity of surface mechanical properties (Figure 11, Figures S10 and S11) might correspond to a slightly higher surface roughness and thus an increase of hydrophobicity (e.g., [63]), but full clarification requires more detailed studies. The observed increase of the polar component may be related to the presence of silsesquioxane structures on the surface. In the case of B-COOMe, the free surface energy is comparable to that of neat components and PLA/PMMA, which is another indicator of a very good blending of components.

## 4. Conclusions

The PLA/PMMA blends modified with 1–3 wt.% of functionalized linear polysilsesquioxanes (LPSQ-R) were partially miscible and phase separated. The obtained results show that small contents of LPSQ-R may improve the dispersion of PMMA in PLA and modulate the properties of such ternary blends. PMMA in the blends formed inclusions, as revealed by SEM. SEM and SEM-EDS demonstrated that LPSQR-R were accumulated in inclusions but were also dispersed in blend polymers on a molecular level, which was accompanied by a slight decrease of *T*_g_ of PLA rich phase. Interestingly, many LPSQ-R containing inclusions were located inside PMMA rich domains, especially in the blends with LPSQ-COOMe and LPSQ-F5. Raman analysis evidenced that the addition of LPSQ-F5 and LPSQ-COOMe resulted in the improved dispersion of PMMA in the blends. The tensile test of the blends demonstrated the improved drawability of PLA/PMMA blend in comparison to neat PLA. Further increase of the drawability was achieved for the blends with the small contents of LPSQ-OH and LPSQ-COOMe, resulting in nearly two-fold increase of elongation at break without a decrease of the yield strength.

## Figures and Tables

**Table 1:** Component contents in the prepared blends.

Sample Code	Component Content		
	PLA (wt.%)	PMMA (wt.%)	LPSQ-R (wt.%)
B-0	90.0	10.0	-
B-R-10	89.1	9.9	1.0
B-R-20	88.2	9.8	2.0
B-R-30	87.4	9.7	2.9

R = OH, COOMe, F5.

## Data Availability

Additional data are available on request.

## Supplementary Materials

The following are available online at https://www.mdpi.com/article/10.3390/polym13071033/s1, Table S1: Summary of the results of SEM analysis. Figure S1: Raman spectra of PLA and PMMA. Table S2: characteristic Raman shifts (cm^−1^) in PLA and LPSQ-R and their assignments. Figure S2: Raman maps illustrating distribution of PMMA and selected spectra of B-0 and B-OH blends. Figure S3: Raman maps illustrating distribution of PMMA and selected spectra of B-0 and B-F5 blends. Figure S4: Raman maps illustrating distribution of PMMA and selected spectra of B-0 and B-COOMe blends. Figure S5: DSC thermograms of neat PLA, PMMA and PLA/PMMA blends modified with LPSQ-R recorded during the first heating at 10 °C min^−^^1^. Figure S6: DSC thermograms of neat PLA, PMMA and PLA/PMMA blends modified with 3 wt.% of LPSQ-R recorded during the first cooling at 10 °C min^−1^. Figure S7: exemplary TGA traces of B-OH and B-COOMe blends in N_2_ at heating rate 10 °C/min. Table S3: yield stress, stress and elongation at break of PLA, PLA/PMMA and LPSQ-R modified PLA/PMMA blends. Figure S8: PLM micrograph of the neck formed in PLA/PMMA specimen during drawing taken after fracture at elongation of approx. 110% (drawing direction horizontal). Figure S9: PLM micrograph of the neck formed in B-COOMe-10 specimen during drawing taken after fracture at elongation of approx. 200% (drawing direction horizontal). Figure S10: maps of hardness (*H*) in compression moulded films of the neat components and the blends (A: surface area; C: cross-section, middle area). Figure S11: maps of elastic modulus (*E*) in compression moulded films of the neat components and the blends (A: surface area; C: cross-section, middle area).
