# Supplementary material for: Phase Structure and Properties of Ternary Polylactide/Poly(methyl methacrylate)/Polysilsesquioxane Blends
Source: Polymers (Basel). 2021 Mar 26;13(7):1033. doi: 10.3390/polym13071033 (PMC8036706; doi:10.3390/polym13071033)
Supplement: Supplementary file 1 [file polymers-13-01033-s001.pdf]

Electronic Supplementary Information

Phase structure and properties of ternary PLA/PMMA/polysilsesquioxane blends.

Anna Kowalewska*, Agata S.Herc, Joanna Bojda, Maria Nowacka, Mariia Svyntkivska, Ewa Piorkowska, Witold Kaczorowski, Witold Szymański

Table S1. Summary of the results of SEM analysis.

**Figure 2**	Cryo-fracture surface of PLA/PMMA (B-0) blend. Inclusions in matrix visible in micrographs, evidencing phase separation.
**Figure 3**	Etched PLA/PMMA (B-0) blend. PMMA-containing particles exposed by etching visible in micrographs.
**Figure 4**	Cryo-fracture surfaces of ternary blends: B-OH-10, B-OH-30, B-F5-10, B-F5-30, B-COOMe-10 and B-COOMe-30. Two types of inclusions in matrices visible, evidencing phase separation.
**Figure 5**	Etched blends: B-OH-30, B-F5-30 and B-COOMe-30. PMMA-containing particles exposed by etching visible in micrographs.

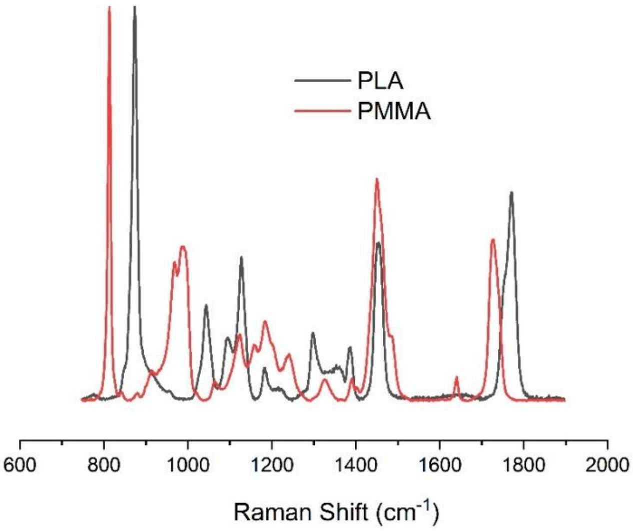

Figure S1. Raman spectra of PLA and PMMA.

Table S2. Characteristic Raman shifts (cm^{-1}) in PLA and LPSQ-R and their assignments.

**PLA** [1]		**PMMA** [2]	
**mode**	**Raman shift (cm^{-1})**	**mode**	**Raman shift (cm^{-1})**
v(C=O)	1767	v(C=O)	1726
$\delta_{as}CH_3$	1452	δ_{as}(C-H) of α-CH_3, δ_{as}(C-H) of O-CH_3	1450
$\delta_{s}CH_3$	1384		
δ_1CH + $\delta_{s}CH_3$	1349		
δ_2CH	1298		
δ(CH) + v(COC)	1220	v(C-O), v(C-COO)	1240
v_{as}(COC) + $r_{as}CH_3$	1180		
$r_{as}CH_3$	1126		
v_s(COC)	1090	v(C-C) skeletal mode	1081
r(C-CH_3)	1042	O-CH_3 rock	990
rCH_3 + vCC	950	v(CH_2)	912
v(C-COO)	873	v(CH_2)	813

[1] Kister, G.; Cassanas, G.; Vert, M. Effects of morphology, conformation and configuration on the IR and Raman spectra of various poly(lactic acid)s. *Polymer* **1998**, *39*, 267-273. https://doi.org/10.1016/S0032–3861(97)00229-2

[2] Bruckmoser, K.; Resch, K.; Kisslinger, T.; Lucyshyn, T. Measurement of interdiffusion in polymeric materials by applying Raman spectroscopy. *Polym. Test.* **2015**, *46*, 12-133. https://doi.org/j.polymertesting.2015.07.004

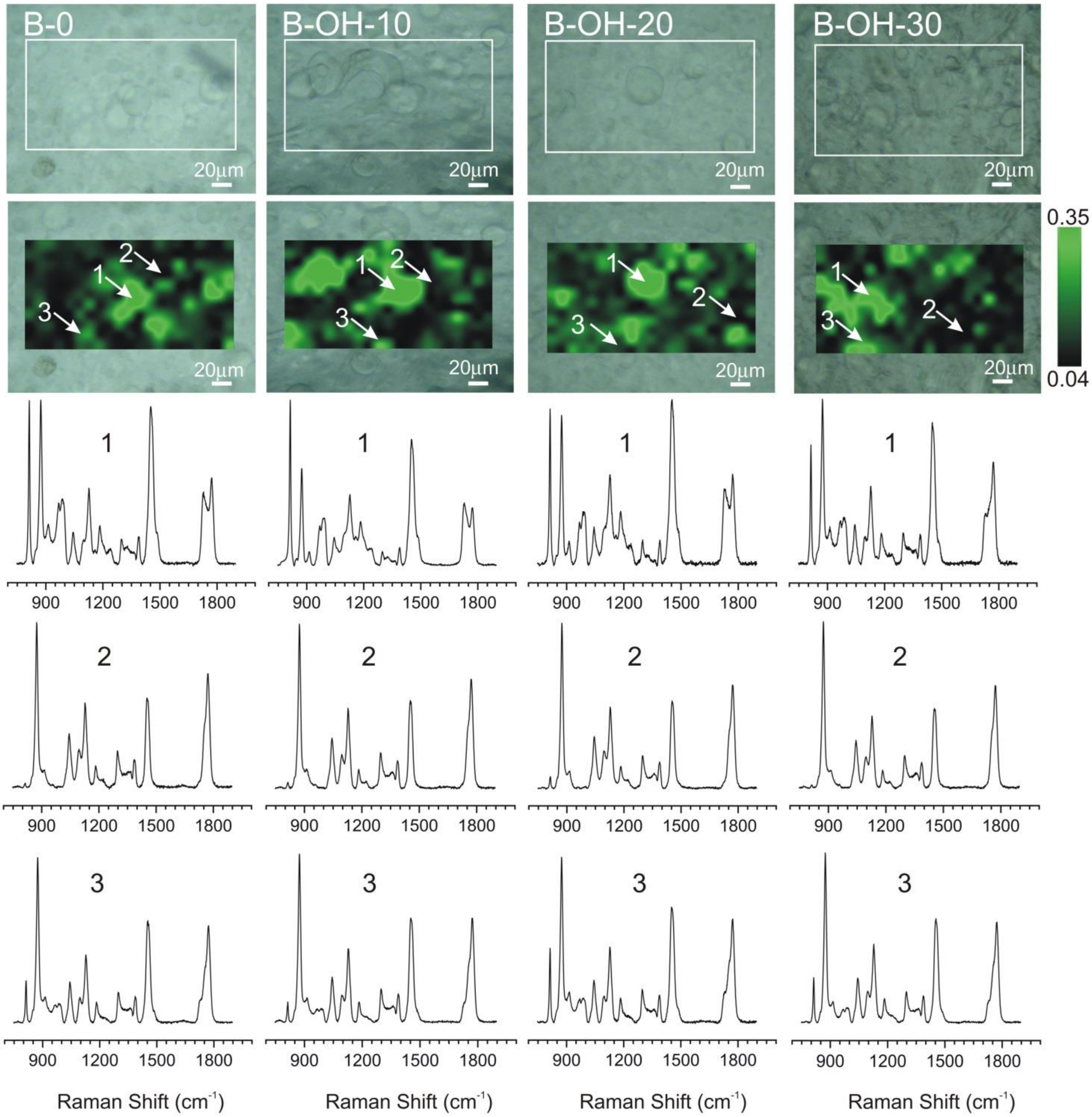

Figure S2. Raman maps illustrating distribution of PMMA and selected spectra of B-0 and B-OH blends.

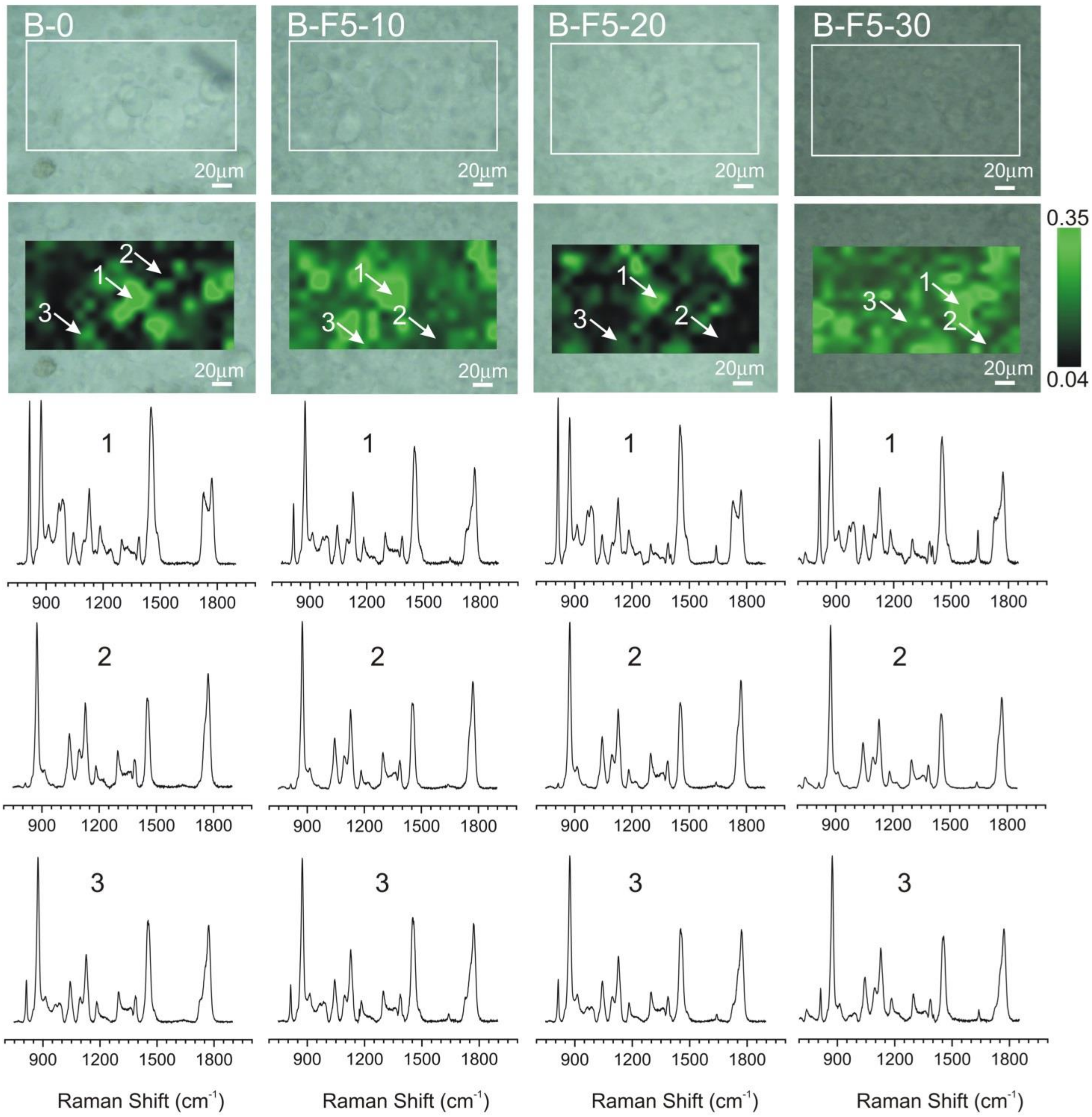

Figure S3. Raman maps illustrating distribution of PMMA and selected spectra of B-0 and B-F5 blends.

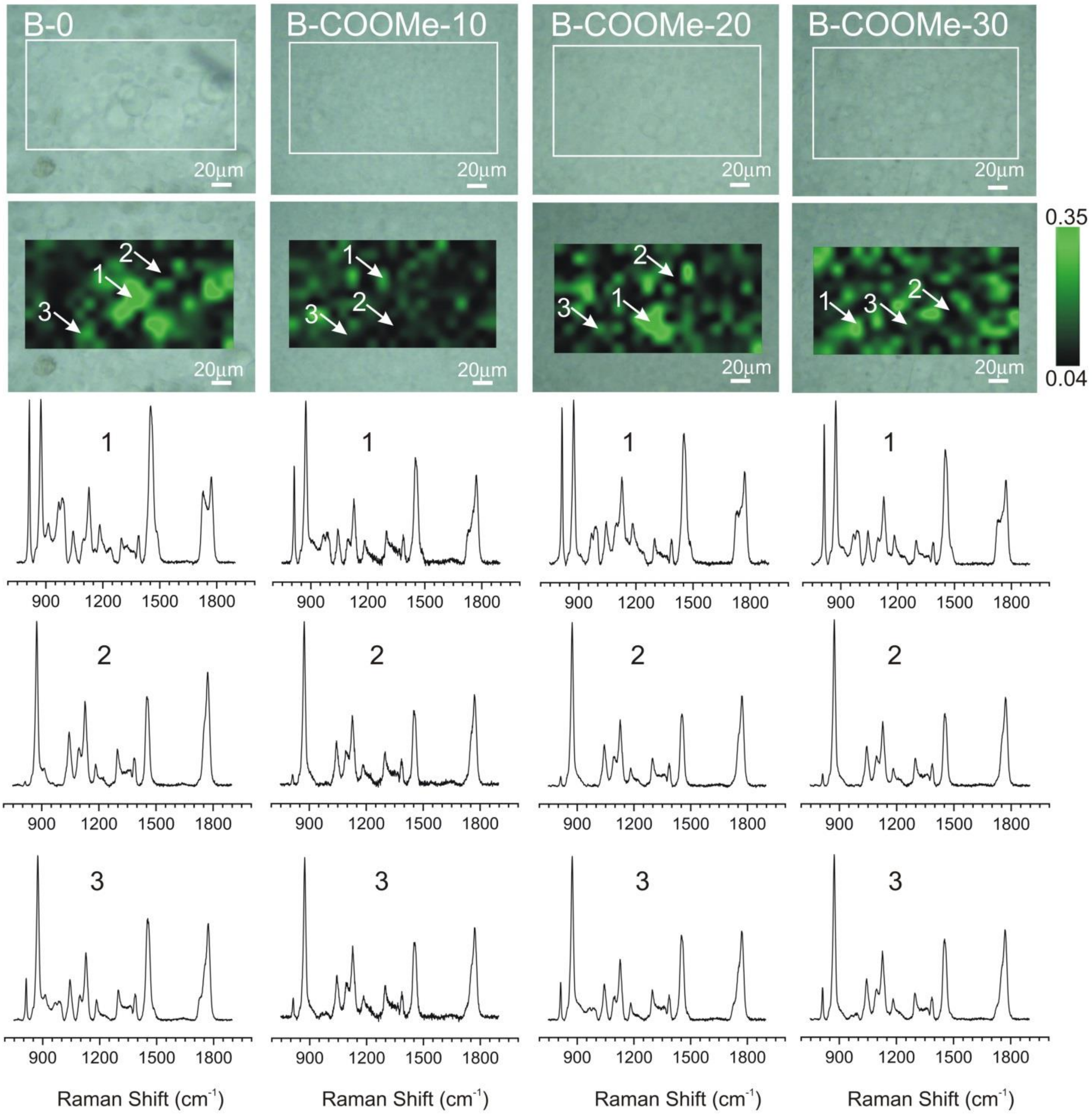

Figure S4. Raman maps illustrating distribution of PMMA and selected spectra of B-0 and B-COOMe blends.

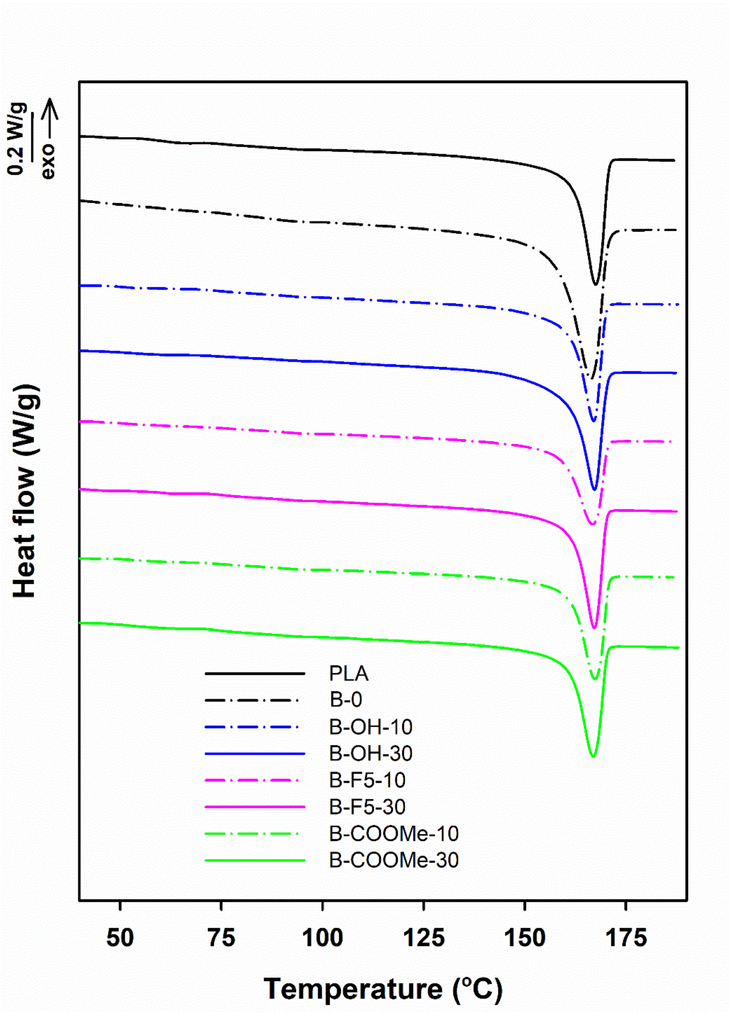

Figure S5. DSC thermograms of neat PLA, PMMA, and PLA/PMMA blends modified with LPSQ-R recorded during the first heating at 10 °C min^{-1}.

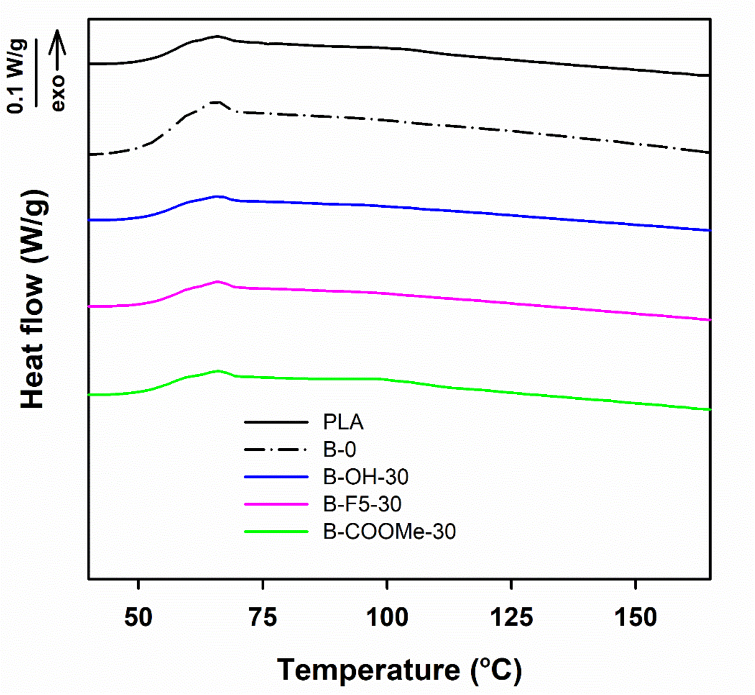

Figure S6. DSC thermograms of neat PLA, PMMA, and PLA/PMMA blends modified with 3 wt.% of LPSQ-R recorded during the first cooling at 10 °C min^{-1}.

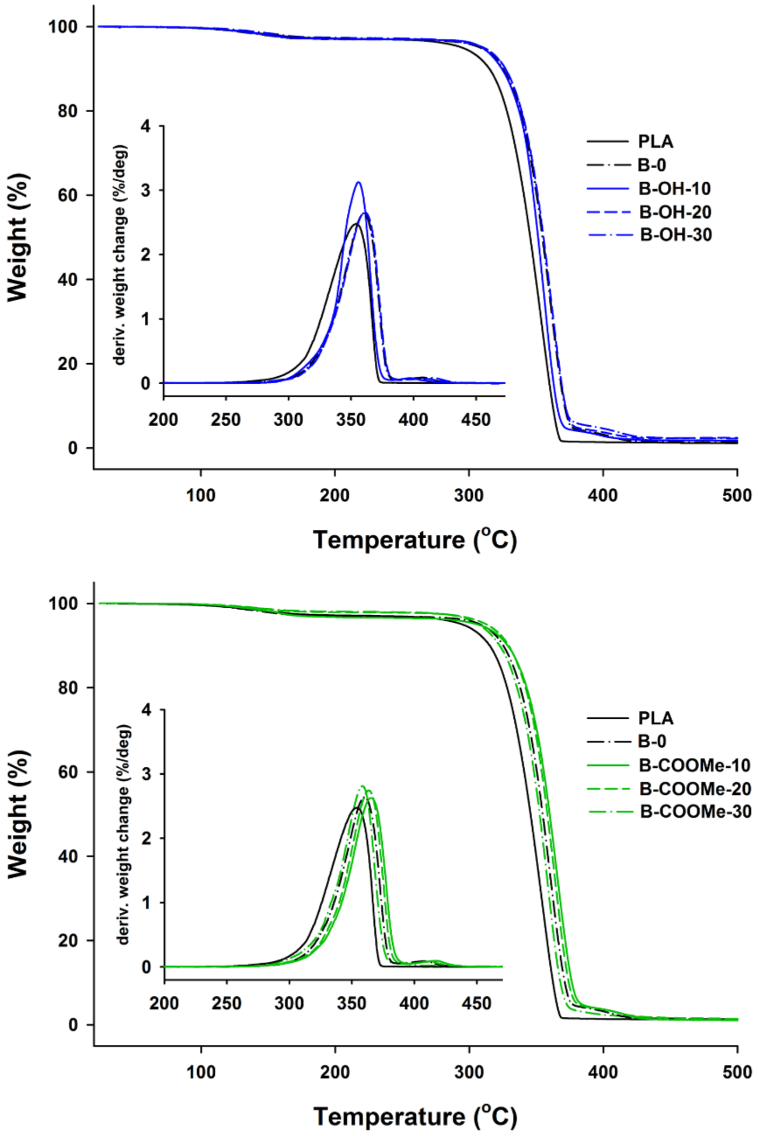

Figure S7. Exemplary TGA traces of B-OH and B-COOMe blends in N_2 at heating rate 10 °C/min.

Note: The small weight loss near 150-160 °C on TGA thermograms results most possibly from sublimation of cyclic oligomers of low molecular weight present in PLA.

Table S3. Yield stress, stress and elongation at break of PLA, PLA/PMMA and LPSQ-R modified PLA/PMMA blends.

Sample code	Yield stress (MPa)	Stress at break (MPa)	Elongation at break (%)
PLA	43.5	41.8	27
B-0	46.4	28.5	95
B-OH-10	45.3	27.3	163
B-OH-20	40.0	33.1	14
B-OH-30	39.0	33.7	11
B-F5-10	43.6	26.0	62
B-F5-20	43.9	27.8	74
B-F5-30	44.7	40.9	11
B-COOMe-10	47.7	28.6	193
B-COOMe-20	48.3	29.1	111
B-COOMe-30	49.0	27.6	49

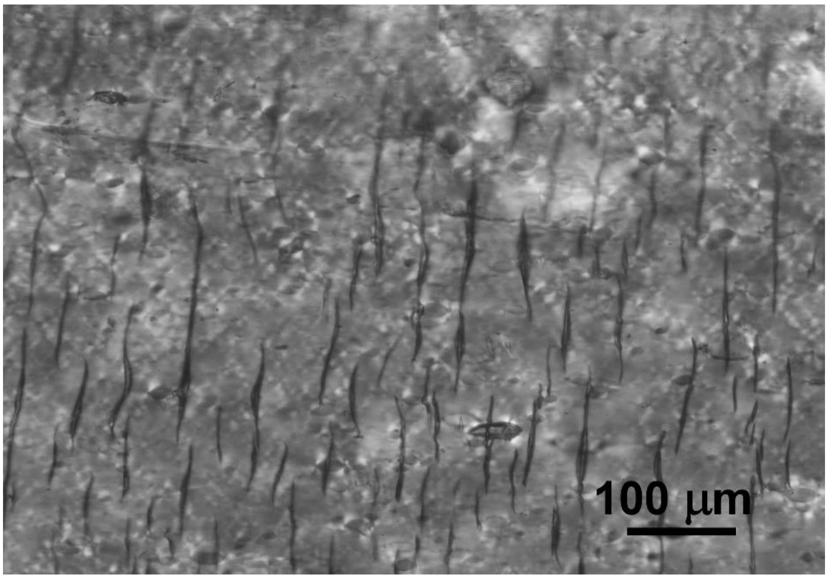

Figure S8. PLM micrograph of the neck formed in PLA/PMMA specimen during drawing, taken after fracture at elongation of approx.110 %. Drawing direction horizontal.

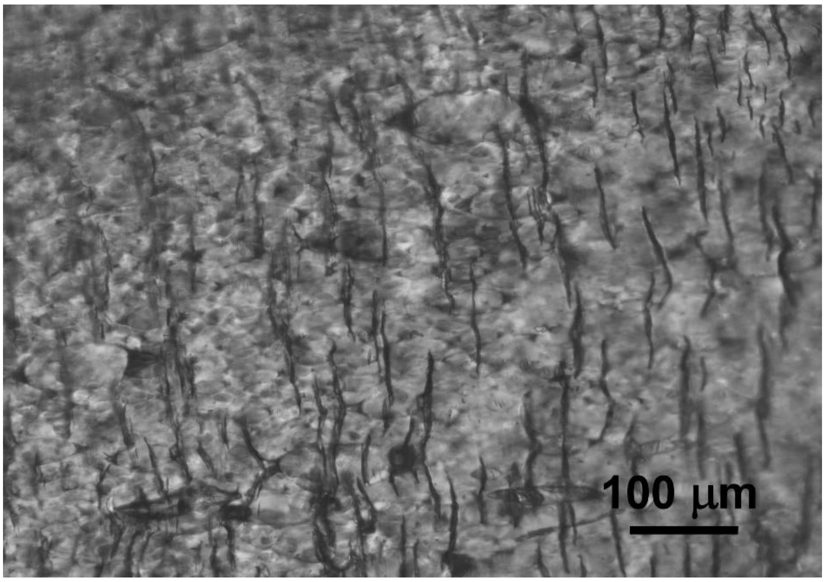

Figure S9. PLM micrograph of the neck formed in B-COOMe-10 specimen during drawing taken after fracture at elongation of approx. 200%. Drawing direction horizontal.

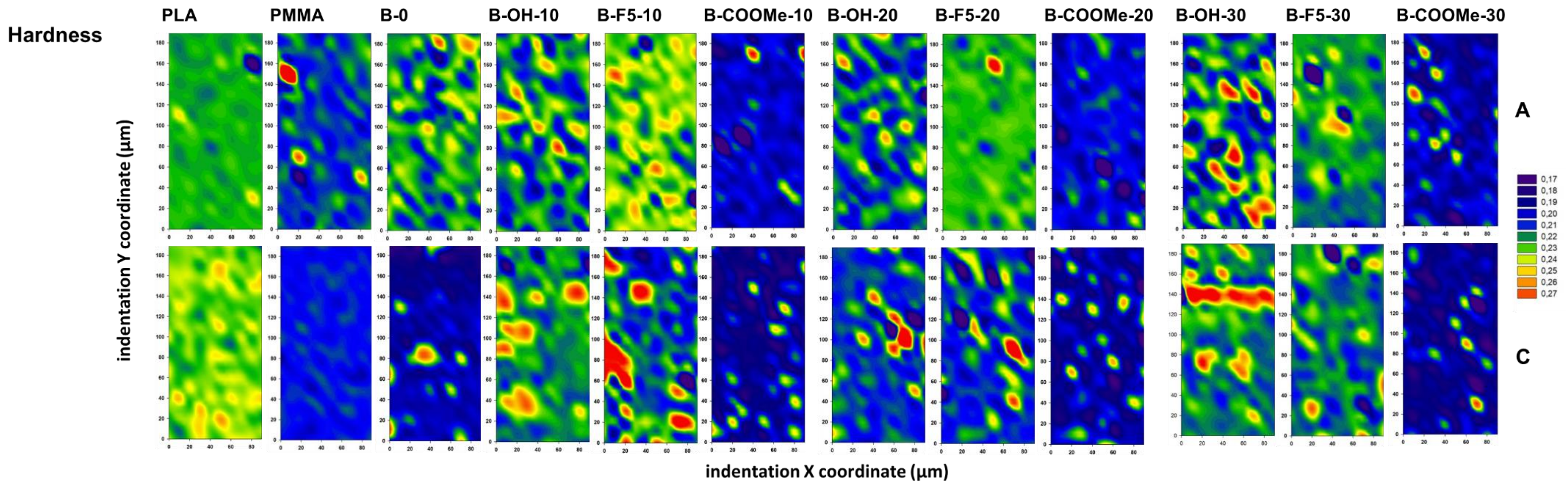

Figure S10. Maps of hardness (*H*) in compression moulded films of the neat components and the blends (A: surface area; C: cross-section, middle area).

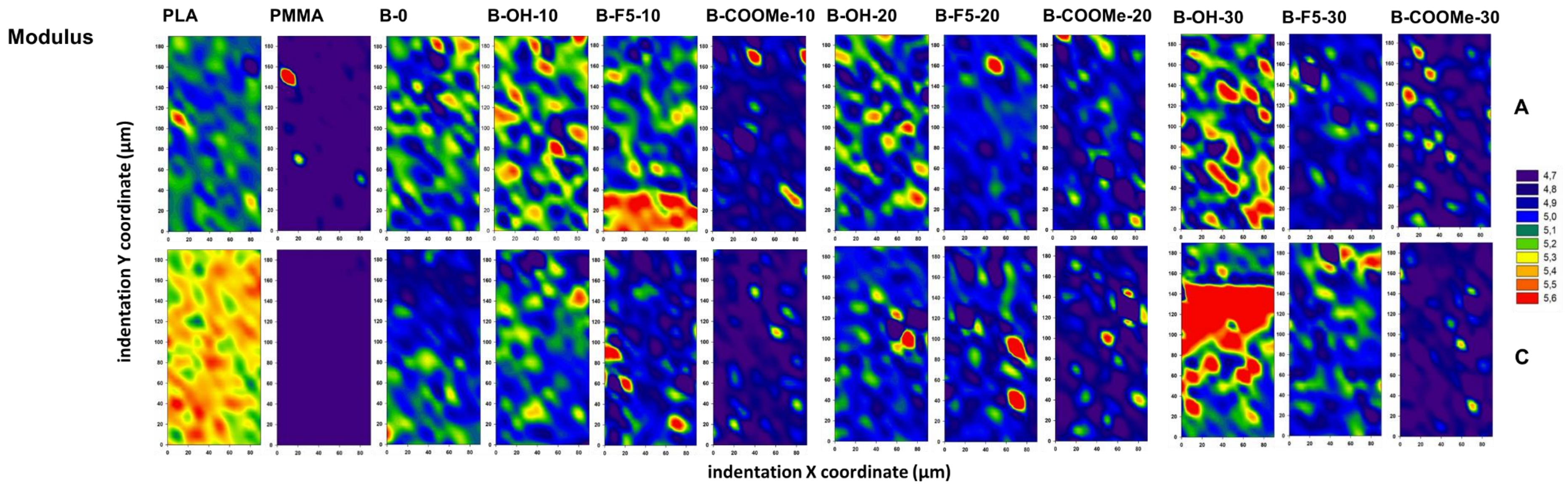

Figure S11. Maps of elastic modulus (*E*) in compression moulded films of the neat components and the blends (A: surface area; C: cross-section, middle area).
